# New molecular sequence data and species trees for North American whipsnakes

**DOI:** 10.1016/j.dib.2018.04.067

**Published:** 2018-04-27

**Authors:** Kyle A. O’Connell, Eric N. Smith

**Affiliations:** a Department of Biology, The University of Texas at Arlington, Arlington, TX 76019, USA; b Department of Vertebrate Zoology; National Museum of Natural History, District of Columbia, WA 20007, USA

## Abstract

In this data article we present species trees based on coalescent species delimitation results for North American whipsnakes, as well as metadata pertaining to the article "The effect of missing data on coalescent species delimitation and a taxonomic revision of whipsnakes (Colubridae: Masticophis)" (MPE-2017-76-R1). Species trees were constructed using SNP data generated from double-digest RADseq, filtered to 80% completeness between species. Tables correspond with the primary manuscript and serve as a repository of genetic sequence information for whipsnakes. These data can be downloaded and combined with future whipsnake datasets.

**Specifications table**

**Table:** 

Subject area	*Biology*
More specific subject area	*Phylogenetics, Herpetology*
Type of data	*Figure, table*
How data was acquired	*Phylogenetic analyses*
Data format	*Analyzed, raw*
Experimental factors	
Experimental features	*Conducted phylogenetic analyses*
Data source location	*USA, Mexico*
Data accessibility	GenBank (KT713652-KT713738), NCBI Short Read Archive SRS1047296, SRS1047267, SRS1047268, SRS1047265

**Value of the data**

• The mitochondrial phylogeny shows the phylogenetic structure of whipsnakes, with an emphasis on the Great Plains lineage of the United States.
• The included species trees reveal previously unknown phylogenetic relationships among whipsnakes.
• Supplementary Table 1 shows metadata for whipsnake sequence data that could be used in future studies.
• Table 1 explains missing data thresholds used for various analyses.

## 1 Data

The data included in this DIB article includes details on two species trees of North American whipsnakes, as well as the meta-data for all whipsnake sequence data used in the original article [1]. The species trees were estimated using coalescent methods from SNP data, with 20% missing data thresholds. Two species trees to encompass both species complexes included in this study. Meta-data includes information for both mitochondrial sequence data, and ddRADseq short sequence reads (Table 1).

**Table 1:** Parameters for each SNP dataset utilized in this study, including the number of loci, and the percent missing data.

Dataset	*N*	% Missing at locus	Mean % missing individual	# Loci	Analysis used
A	14	30	20	365	SPLITSTREE
B	26	50	35.3	2077	SNAPP
C	26	20	13.3	325	SNAPP
D	10	50	38.6	1464	SNAPP
E	10	20	16.2	216	SNAPP

## 2 Experimental design, materials and methods

### 2.1 Mitochondrial phylogenetic analysis

**Fig. 1:**
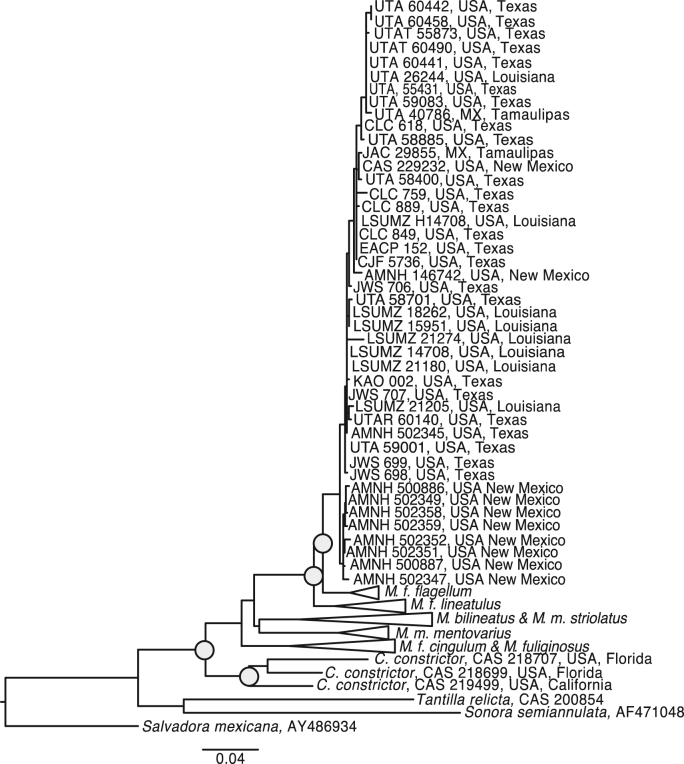
Maximum likelihood phylogeny generated from mtDNA. The full clade representing *Masticophis flagellum testaceus* is shown. All other clades are collapsed. Nodes with at least 70% bootstrap support are shown with grey circles.

We aligned all sequences with the Geneious Aligner under default settings [2]. We calculated uncorrected average pairwise distance between lineages in Mega v7 [3]. We selected the most probable model of nucleotide evolution for Likelihood analyses using Bayesian information criteria implemented in PartitionFinder [4], partitioning by codon position. We estimated a maximum likelihood phylogeny using raxmlGUI v1.3 with 1000 rapid bootstrap iterations [5] and visualized our final phylogeny in FigTree v1.4.3 [6]. We considered nodes with bootstrap values ≥70 as strongly supported (Fig. 1).

### 2.2 SNP-based species tree analysis

**Fig. 2:**
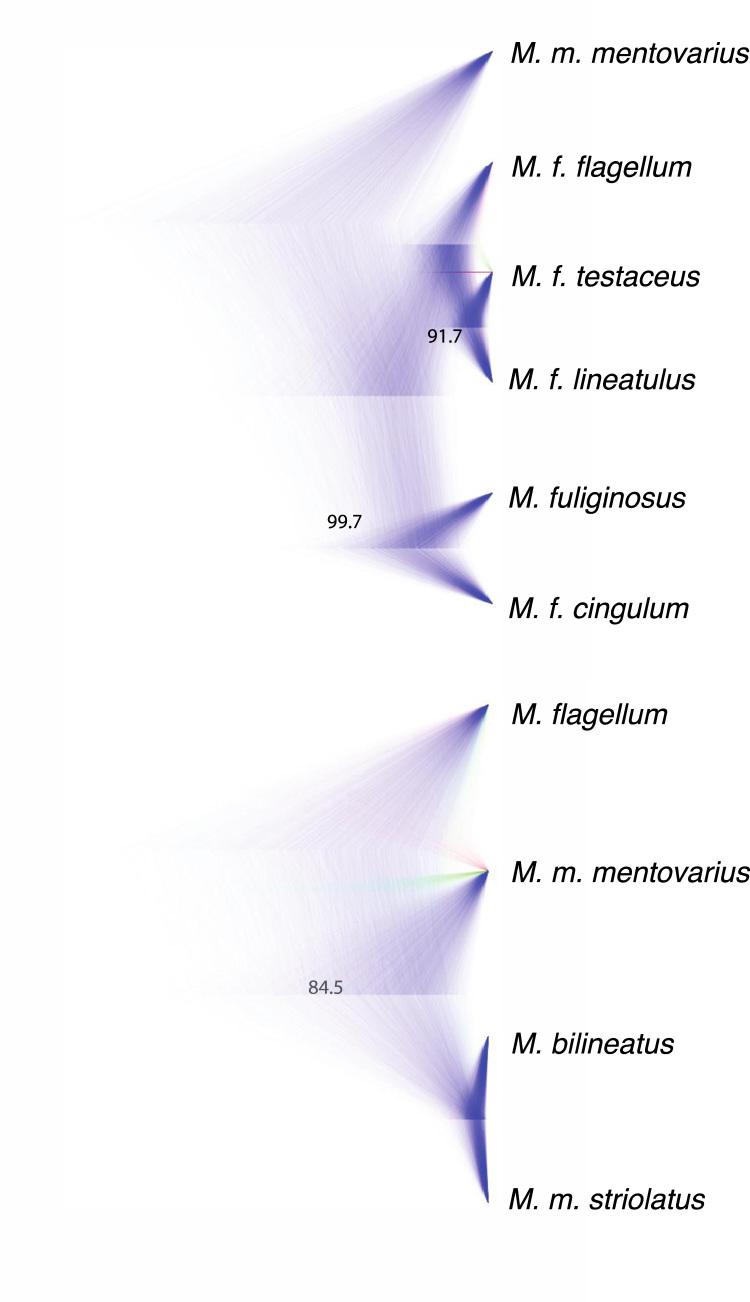
Species trees generated using SNAPP based on the best-supported models from our Bayes Factor delimitation analysis from the primary manuscript for datasets C and E (< 20% missing loci). Support values are labeled for each node that is not fully supported.

Species trees were estimated with ≤20% missing data using SNAPP v1.0. We assigned species identities based on the best supported model from our BFD* analyses. We allowed BEAUti to estimate the mutation rate, and confirmed that both U and V were approximately equal to one. We assigned a Gamma distribution to our Lambda prior, with an Alpha of 1 and a Beta of 77. On our Snap prior we assigned an Alpha of 1, a Beta of 100, and a Lambda of 77. We ran the analyses for 10,000,000 MCMC generations, sampling every 1000 generations. We visualized the complete tree sets in DENSITREE v1.0 [7], and removed the first 10% of trees as burn-in (Fig. 2).
