# Supplementary material for: New molecular sequence data and species trees for North American whipsnakes
Source: Data Brief. 2018 Apr 27;18:1995–9. doi: 10.1016/j.dib.2018.04.067 (PMC5998179; doi:10.1016/j.dib.2018.04.067)
Supplement: Supplementary file 2 — Supplementary material [file mmc2.docx]

Table 1: Locality and online repository information for all whipsnake samples used in this study.

| Specimen Number | Species | Country | State: Locality | Latitude | Longitude | GenBank ID | SRA | Source |
| --- | --- | --- | --- | --- | --- | --- | --- | --- |
| UTA 57969 (JAC 30222) | *bilineatus* | Mexico | Nayarit: Road W of Mesquites | 20.9279 | -104.5495 | KY007695 |  | O'Connell et al., 2017 |
| ROM 14965 | *bilineatus* | Mexico | Sinaloa: North west of Fresnilla: West of HW 45 | 23.37 | -103.36 | KT713628 |  | O'Connell et al., 2017 |
| UTA 23306 | *bilineatus* | Mexico | Jalisco: HW 23 Tlaltenagno de Sanchez Roman- Colotlan | 22.03587 | -103.26819 | KT713726 | SRS1047269 | O'Connell et al., 2017 |
| MVZ 225550 | *bilineatus* | USA | Arizona: Cochise | 31.9136 | -109.144 | KP765657 |  |  |
| JAC 30654 | *flagellum* | Mexico | Sonora | 27.12109 | -109.517115 | KT713652 |  | O'Connell et al., 2017 |
| AMNHR 139223 | *flagellum* | USA | Arizona: Cochise | 31.95 | -109.97 | KT713629 |  | O'Connell et al., 2017 |
| AMNHR 146742 | *flagellum* | USA | New Mexico: HW 9: Between Animas and Windmill | 31.95 | -108.73 | KT713630 |  | O'Connell et al., 2017 |
| CAS 195954 | *flagellum* | USA | Florida: HW 41 S Florida Ave | 28.69 | -82.33 | KT713631 |  | O'Connell et al., 2017 |
| CAS 214850 | *flagellum* | USA | Florida: Avon Park: HW 27 | 27.541194 | -81.48825 | KT713632 | SRS1047331 | O'Connell et al., 2017 |
| CAS 229232 | *flagellum* | USA | New Mexico: South of Conchas: HW 104 | 35.344667 | -104.219167 | KT713633 | SRS1047326 | O'Connell et al., 2017 |
| CAS 229237 | *flagellum* | USA | New Mexico: HW 11 south of Deming | 32.124 | -107.7515 | KT713634 | SRS1047325 | O'Connell et al., 2017 |
| CAS 231705 | *flagellum* | USA | Florida: North of Woodville | 30.34 | -84.25 | KT713635 | SRS1047324 | O'Connell et al., 2017 |
| CJF 5736 | *flagellum* | USA | Texas: East of Fort McKavett: South of Hw 190 | 30.863233 | -100.030272 | KT713636 |  | O'Connell et al., 2017 |
| UTA 58400 (CLC 63) | *flagellum* | USA | Texas: North of Alice: HW 3376 | 27.79479 | -98.04662 | KT713637 |  | O'Connell et al., 2017 |
| UTA 58885 (CLC 531) | *flagellum* | USA | Texas | 27.60302 | -98.41345 | KT713638 |  | O'Connell et al., 2017 |
| CLC 618 | *flagellum* | USA | Texas: Old Decatur Rd | 33.307 | -97.60629 | KT713639 | SRS1047317 | O'Connell et al., 2017 |
| CLC 759 | *flagellum* | USA | Texas: HW 352 and HW 147 | 32.6166 | -99.4666 | KT713640 |  | O'Connell et al., 2017 |
| CLC 849 | *flagellum* | USA | Texas: Beeville: HW 351 | 28.38935 | -97.77164 | KT713641 |  | O'Connell et al., 2017 |
| CLC 889 | *flagellum* | USA | Texas: South of Alice: West of HW 281 | 27.60302 | -98.14345 | KT713642 |  | O'Connell et al., 2017 |
| FTB 840 | *flagellum* | USA | Georgia: South of Atlanta: HW 75 | 33.6 | -84.28 | KT713644 |  | O'Connell et al., 2017 |
| FTB 1142 | *flagellum* | USA | Georgia: East of Eastman: HW 46 | 32.22 | -82.99 | KT713645 | SRS1047303 | O'Connell et al., 2017 |
| FTB 2451 | *flagellum* | USA | Georgia: South of Atlanta: HW 75 | 33.6 | -84.28 | KT713646 |  | O'Connell et al., 2017 |
| LSUMZ H-14708 | *flagellum* | USA | Louisiana: Kisatchie National Park: HW 167 | 31.736 | -92.577 | KT713647 |  | O'Connell et al., 2017 |
| JAC 27587 | *flagellum* | Mexico | Michoachan | 19.6986 | -102.185 | KT713648 |  | O'Connell et al., 2017 |
| UTA 57968 (JAC 29855) | *flagellum* | Mexico | Tamaulipas: HW 80: North of Cuauhtemoc | 22.587148 | -98.21237 | KT713649 | SRS1047295 | O'Connell et al., 2017 |
| UTA 57751 (JAC 30567) | *flagellum* | Mexico | Sonora: Navojoa: HW 15 | 27.12109 | -109.517115 | KT713650 | SRS1047293 | O'Connell et al., 2017 |
| UTA 57752 (JAC 30568) | *flagellum* | Mexico | Sonora: Navojoa: HW 15 | 27.12109 | -109.517115 | KT713651 | SRS1047292 | O'Connell et al., 2017 |
| JMM 778 | *flagellum* | Mexico | Baja California: HW 5 San Felipe- Mexicali: South of Colonia la Puerta | 32.308442 | -115.324732 | KT713653 |  | O'Connell et al., 2017 |
| UTA 59001 (JWS 041) | *flagellum* | USA | Texas: Fort Davis: HW 17 | 30.605 | -103.87533 | KT713654 | SRS1047291 | O'Connell et al., 2017 |
| UTA 58701 (JWS 261) | *flagellum* | USA | Texas: North of Abilene on HW 351 | 32.6547 | -99.45863 | KT713655 |  | O'Connell et al., 2017 |
| JWS 698 | *flagellum* | USA | Texas: East of Pecos: HW 20 | 31.4 | -103.45 | KT713656 |  | O'Connell et al., 2017 |
| JWS 699 | *flagellum* | USA | Texas: East of Pecos: HW 20 | 31.4 | -103.45 | KT713657 |  | O'Connell et al., 2017 |
| JWS 706 | *flagellum* | USA | Texas: East of Pecos: HW 20 | 31.4 | -103.45 | KT713658 |  | O'Connell et al., 2017 |
| JWS 707 | *flagellum* | USA | Texas: South of llano: South west of Horseshoe Bay | 30.45 | -98.5 | KT713659 |  | O'Connell et al., 2017 |
| KAO 002 | *flagellum* | USA | Texas: South West of San Angelo: East of 163 | 30.997 | -101.054 | KT713660 |  | O'Connell et al., 2017 |
| LSUMZ H-2451 | *flagellum* | USA | Florida: Munson Recreation Area: HW 4 | 30.8559 | -86.853 | KT713661 | SRS1047277 | O'Connell et al., 2017 |
| LSUMZ H-8153 | *flagellum* | USA | Alabama: East of Oyster Bay | 30.261 | -87.709 | KT713662 |  | O'Connell et al., 2017 |
| LSUMZ H-15951 | *flagellum* | USA | Louisiana: Lime Kiln Rd | 31.6799 | -93.1779 | KT713664 |  | O'Connell et al., 2017 |
| LSUMZ H18262 | *flagellum* | USA | Louisiana: Sabine Parish: East of Sabine National Forest | 31.42 | -93.582 | KT713665 |  | O'Connell et al., 2017 |
| LSUMZ H-18425 | *flagellum* | USA | New Mexico: HW 9 and 338 | 31.949675 | -108.806706 | KT713666 |  | O'Connell et al., 2017 |
| LSUMZ H-19717 | *flagellum* | USA | Florida: East of HW 98, West of Citrus Wildlife Management Area | 28.789 | -82.495 | KT713667 |  | O'Connell et al., 2017 |
| LSUMZ H-21180 | *flagellum* | USA | Louisiana: East of Kepler Creek Lake on Piney Woods Rd | 32.335 | -93.1233 | KT713668 | SRS1047280 | O'Connell et al., 2017 |
| LSUMZ H-21205 | *flagellum* | USA | Louisiana: West of Bienville: on Bp 699 | 32.408 | -93.022 | KT713669 |  | O'Connell et al., 2017 |
| LSUMZ H21274 | *flagellum* | USA | Louisiana: Kepler Creek Lake | 32.32 | -93.116 | KT713670 | SRS1047278 | O'Connell et al., 2017 |
| MVZ 161425 | *flagellum* | Mexico | Baja California: HW 1 Lazaro Cardenas- Guerrero Negro | 28.515833 | -114.03 | KT713671 | SRS1047273 | O'Connell et al., 2017 |
| UTAR 60140 | *flagellum* | USA | Texas: Crescent Heights: West of 753 | 32.169 | -95.947 | KT713673 |  | O'Connell et al., 2017 |
| ROM 14197 | *flagellum* | MX | Sonora: HW 8 Puerto Penasco- Sonoyta | 31.39 | -113.5 | KT713674 |  | O'Connell et al., 2017 |
| ROM 14948 | *flagellum* | MX | Chihuahua: North of El Bachivo: South of HW 13 Carr Navojoa - Alamos | 27.07 | -109.32 | KT713675 |  | O'Connell et al., 2017 |
| ROM 15050 | *flagellum* | MX | Sonora: West of Hermosillo: HW 100 | 28.8 | -111.91 | KT713676 | SRS1047243 | O'Connell et al., 2017 |
| UTAR 26244 | *flagellum* | USA | Texas: North of Decatur | 33.391 | -97.55 | KT713677 |  | O'Connell et al., 2017 |
| UTAT 40786 | *flagellum* | Mexico | Tamaulipas: HW 97 East of Monterrey | 25.3 | -98.305655 | KT713678 |  | O'Connell et al., 2017 |
| UTAR 55431 | *flagellum* | USA | Texas: South of Lake Whitney State Park | 31.79 | -97.42 | KT713679 |  | O'Connell et al., 2017 |
| UTAT 59083 | *flagellum* | Mexico | Tamaulipas: HW 97 East of Monterrey | 25.3 | -98.305655 | KT713680 |  | O'Connell et al., 2017 |
| UTAT 60441 | *flagellum* | USA | Texas: North of Comanche: HW 16 | 31.93427 | -98.575658 | KT713681 | SRS1047249 | O'Connell et al., 2017 |
| UTAT 60442 | *flagellum* | USA | Texas: West of Kileen: South of Colorado Bend State Park | 30.987421 | -98.575658 | KT713682 |  | O'Connell et al., 2017 |
| UTAR 60458 | *flagellum* | USA | Texas: East of Laredo: West of 16 | 27.253 | -98.94 | KT713683 |  | O'Connell et al., 2017 |
| UTAT 55873 | *flagellum* | USA | Texas: South of Antelope: Prideaux Rd | 33.40475 | -98.40544 | KT713684 |  | O'Connell et al., 2017 |
| UTAR 60490 | *flagellum* | USA | Texas |  |  | KT713685 |  | O'Connell et al., 2017 |
| ANF I5 | *flagellum* | USA | Texas: Angelia | 31.145794 | -94.709484 |  |  | O'Connell et al., 2017 |
| ANF I7 | *flagellum* | USA | Texas: Angelia | 31.145794 | -94.709484 |  |  | O'Connell et al., 2017 |
| ASH 177 | *flagellum* | USA | Texas: Big Bend Ranch State Park | 29.49495 | -103.8988833 |  |  | O'Connell et al., 2017 |
| ASH 222 | *flagellum* | Mexico | Coahuila: Rancho El Salado | 29.33978333 | -102.6546667 |  |  | O'Connell et al., 2017 |
| CJF 5816 | *flagellum* | USA | Arizona: South west of Phoenix: HW 8 | 32.701 | -113.75992 |  |  | O'Connell et al., 2017 |
| UTEP 20749 (CSL 9485) | *flagellum* | USA | Texas: North of Fort Davis: HW 118 | 30.726954 | -104.131703 |  |  | O'Connell et al., 2017 |
| UTEP 18542 (CSL 9506) | *flagellum* | USA | New Mexico: Lincoln National Forest: Cloudcroft | 32.96032 | -105.685018 |  | SRS1047310 | O'Connell et al., 2017 |
| UTEP 20772 (CSL 9511) | *flagellum* | USA | Texas: North of Fort Davis: HW 118 | 30.726954 | -104.131703 |  |  | O'Connell et al., 2017 |
| DRS 011 | *flagellum* | USA | Texas: North west of Kileen: Blakely Rd | 31.33527 | -98.09553 |  |  | O'Connell et al., 2017 |
| JAC 29104 | *flagellum* | Mexico | Chihuahua: HW 24 | 26.242652 | -106.518098 | KY007697 | SRS1047297 | O'Connell et al., 2017 |
| JWS 709 | *flagellum* | USA | Texas: East of Pecos: HW 20 | 31.4 | -103.45 |  | SRS1047288 | O'Connell et al., 2017 |
| LSUMZ H-20959 | *flagellum* | USA | Louisiana: North west of Bogalusa, south of Dean Lee State Forest | 30.8375 | -89.95 |  | SRS1047281 | O'Connell et al., 2017 |
| MAFL SL5 | *flagellum* | USA | Louisiana: Bienville Parish | 321.25 | -93.265 |  |  | O'Connell et al., 2017 |
| UTA 53316 (24067) | *flagellum* | Mexico | Colima: North of La Boca de Apiza: HW 200 | 18.72513 | -103.72293 |  |  | O'Connell et al., 2017 |
| TCWC 95170 | *flagellum* | USA | Texas: West of College Station | 30.7 | -96.2011 |  |  | O'Connell et al., 2017 |
| JAC 24853 (MX 20-30) | *flagellum* | Mexico | Sonora: Hwy between Hornos and San Nicolas | 27.74358 | -109.76166 | KY007698 |  | Myers et al., 2016 |
| EACP152 | *flagellum* | USA | Texas: Dallas Co. | 32.078 | -96.930922 | KY007696 |  | Myers et al., 2016 |
| AMNH 502345 | *flagellum* | USA | Texas | 32.76217 | -99.0825 | KX835748 |  | Myers et al., 2016 |
| AMNH 502347 | *flagellum* | USA | Texas: west Texas | 32.71121 | -102.58082 | KX835749 |  | Myers et al., 2016 |
| AMNH 502349 | *flagellum* | USA | New Mexico: Near Malaga | 32.1014 | -104.07269 | KX835750 |  | Myers et al., 2016 |
| AMNH 502351 | *flagellum* | USA | Texas: near Pecos | 31.6463 | -103.66731 | KX835751 |  | Myers et al., 2016 |
| AMNH 502352 | *flagellum* | USA | New Mexico: Near Carlsbad | 32.18395 | -103.40491 | KX835752 |  | Myers et al., 2016 |
| AMNH 502358 | *flagellum* | USA | New Mexico: Near Lake Arthur | 32.95682 | -104.38033 | KX835753 |  | Myers et al., 2016 |
| AMNH 502359 | *flagellum* | USA | New Mexico: Near Carlsbad | 32.19728 | -104.33687 | KX835754 |  | Myers et al., 2016 |
| AMNH 502363 | *flagellum* | USA | New Mexico | 33.5808 | -105.96581 | KX835755 |  | Myers et al., 2016 |
| AMNH 502370 | *flagellum* | USA | New Mexico: Near Gila National Forest | 32.60183 | -107.32455 | KX835756 |  | Myers et al., 2016 |
| AMNH 502378 | *flagellum* | USA | New Mexico: Hachita | 31.875 | -108.33517 | KX835757 |  | Myers et al., 2016 |
| AMNH 502409 | *flagellum* | USA | Arizona: Sonora Desert National Monument | 33.059965 | -112.254944 | KX835758 |  | Myers et al., 2016 |
| AMNH 502410 | *flagellum* | USA | Arizona: Sonora Desert National Monument | 33.043203 | -112.322021 | KX835759 |  | Myers et al., 2016 |
| AMNH 502504 | *flagellum* | USA | Arizona: far west | 33.91423 | -114.02694 | KX835760 |  | Myers et al., 2016 |
| AMNH 502420 | *flagellum* | USA | Arizona: Near Wickenburg | 33.93536 | -112.6926 | KX835761 |  | Myers et al., 2016 |
| AMNH 502423 | *flagellum* | USA | Arizona: Dudleyville | 32.93947 | -110.73831 | KX835762 |  | Myers et al., 2016 |
| AMNH 502426 | *flagellum* | USA | Arizona: Hw 79 | 32.74914 | -111.13174 | KX835763 |  | Myers et al., 2016 |
| AMNH 502445 | *flagellum* | USA | Arizona: Apache | 31.65849 | -109.1551 | KX835764 |  | Myers et al., 2016 |
| AMNH 502450 | *flagellum* | USA | New Mexico: Columbus | 31.82879 | -107.6392 | KX835765 |  | Myers et al., 2016 |
| AMNH 502451 | *flagellum* | USA | New Mexico: Alamogordo, near wt sands nat. monument | 32.797448 | -106.134896 | KX835766 |  | Myers et al., 2016 |
| AMNH 500882 | *flagellum* | USA | Arizona: W of Phoenix | 33.2577 | -113.13875 | KX835773 |  | Myers et al., 2016 |
| AMNH 500883 | *flagellum* | USA | Arizona: Near Chiricahua national monument | 31.936722 | -109.135809 | KX835774 |  | Myers et al., 2016 |
| AMNH 500884 | *flagellum* | USA | Arizona: Near Chiricahua national monument | 31.96045 | -109.14418 | KX835771 |  | Myers et al., 2016 |
| AMNH 500885 | *flagellum* | USA | New Mexico, South of Albuquerque | 34.33763 | -106.87869 | KX835775 |  | Myers et al., 2016 |
| AMNH 500886 | *flagellum* | USA | New Mexico, South of Albuquerque | 34.65024 | -106.81205 | KX835776 |  | Myers et al., 2016 |
| AMNH 500887 | *flagellum* | USA | New Mexico, South of Albuquerque | 34.41957 | -106.7651 | KX835777 |  | Myers et al., 2016 |
| AMNH 500889 | *flagellum* | USA | New Mexico: South of Deming | 32.04774 | -107.70973 | KX835769 |  | Myers et al., 2016 |
| AMNH 500890 | *flagellum* | USA | New Mexico: Columbus | 31.82276 | -107.65617 | KX835770 |  | Myers et al., 2016 |
| AMNH 500891 | *flagellum* | USA | New Mexico: near Apache | 31.59724 | -109.23574 | KX835772 |  | Myers et al., 2016 |
| UF 150082 | *flagellum* | USA | Florida | 30.568611 | -85.812222 | KT447218 |  | Myers et al., 2016 |
| MVZ 245881 | *flagellum* | USA | California: Kern County | 35.1245 | -118.16728 | KX835787 |  | Myers et al., 2016 |
| MVZ 234614 | *flagellum* | USA | California: San Bernardino County | 34.90243 | -115.74564 | KX835786 |  | Myers et al., 2016 |
| MVZ 229145 | *flagellum* | USA | California: Kern County | 35.611423 | -118.2426654 | KX835785 |  | Myers et al., 2016 |
| CAS 223614 | *flagellum* | USA | California: San Diego County | 33.1532475 | -116.1584318 | KX835783 |  | Myers et al., 2016 |
| CAS 200662 | *flagellum* | USA | California: Riverside County | 33.675 | -117.0115 | KX835782 |  | Myers et al., 2016 |
| CAS 200381 | *flagellum* | USA | California: San Diego County | 33.0083 | -116.8678167 | KX835781 |  | Myers et al., 2016 |
| CAS 200375 | *flagellum* | USA | California: Riverside County | 33.80055556 | -117.2552778 | KX835780 |  | Myers et al., 2016 |
| JAC 30652 | *flagellum* | MX | Sonora | 27.12109 | -109.517115 | KX835768 |  | Myers et al., 2016 |
| CAS 219734 | *flagellum* | USA | California: Kern County | 35.6485 | -118.3580278 | AY486928 |  | Myers et al., 2016 |
| AMNH 502362 | *flagellum* | USA | New Mexico | 33.5808 | -105.96581 | KX835755 |  | Myers et al., 2016 |
| UTA 29887 (ENS 2669) | *mentovarius* | Guatemala | Escuintla: HW 14 | 14.333 | -90.841939 | KT713697 | SRS1047305 | O'Connell et al., 2017 |
| MVZ 204113 | *mentovarius* | Costa Rica | Alajuela: Montenegro: HW 1 Panamerican HWY | 10.48333 | -85.21667 |  | SRS1047247 | O'Connell et al., 2017 |
| UTA 23750 | *mentovarius* | Mexico | Jalisco: Ambrosio: Close to HW 80 | 20.24336 | -103.99 | KT713719 | SRS1047266 | O'Connell et al., 2017 |
| UTA 52010 (ENS 10132) | *mentovarius* | Guatemala | Zacapa: HW 3: Communidad Santiago | 15.075086 | -89.438964 | KT713699 | SRS1047307 | O'Connell et al., 2017 |
| MX 24271 | *mentovarius* | Mexico | Oaxaca: HW 200 Acapulco - Salina Cruz | 15.72556 | -96.66186 | KT713712 | SRS1047259 | O'Connell et al., 2017 |
| UTAR 53341 (JAC 24305) | *mentovarius* | Mexico | Oaxaca: HW 200 Acapulco - Salina Cruz | 15.73917 | -96.80701 | KT713722 | SRS1047258 | O'Connell et al., 2017 |
| MEX 23720 | sp. | Mexico | Jalisco: Ambrosio: Close to HW 80 | 20.29284 | -103.99 | KT713693 |  | O'Connell et al., 2017 |
| JAC 28128 | sp. | Mexico | Colima:Road from Colima to Minatitlan and HWY 98 from Minatitlan to Manzanillo | 19.21555 | -104.21592 | KY007699 |  | Myers et al., 2016 |
| UTA 57967 (JAC 29377) | sp. | Mexico | Durango: East of la Reserva de la Biosfera de La Michilia: HW 241 | 23.422366 | -104.175217 | KT713692 | SRS1047296 | O'Connell et al., 2017 |
| UTA 23619 | sp. | Mexico | Jalisco: Ambrosio: Close to HW 80 | 20.29284 | -103.99 | KT713694 | SRS1047268 | O'Connell et al., 2017 |
| UTA 53441 (JAC 23753) | sp. | Mexico | Nayarit: Valle Dorado: Blvrd Niviera Nayarit | 20.714 | -105.27647 | KT713695 | SRS1047265 | O'Connell et al., 2017 |
| CAS 218707 | Coluber constrictor | USA | Florida | 29.03526111 | -82.46094 | EU180430 |  | Burbrink et al., 2008 |
| CAS 218699 | Coluber constrictor | USA | Florida | 30.16586111 | -82.2003333 | EU180433 |  | Burbrink et al., 2009 |
| CAS 219499 | Coluber constrictor | USA | California | 39.60305556 | -122.9013611 | EU180466 |  | Burbrink et al., 2010 |
| CAS 200845 | Tantilla relicta | USA | Florida | 28.69115 | -82.33772 | AF471045 |  | Lawson et al., 2005 |
| CAS 206503 | Sonora semiannulata | USA | California | 36.2452 | -117.45315 | AF471048 |  | Lawson et al., 2006 |
| CAS 212760 | Salvadora mexicana | USA | California | 39.16058333 | -122.6680833 | AY486914 |  | Nagy et al., 2004 |
